# Antifungal efficacy of microencapsulated oligoDNAs through whey protein concentrate (WPC) as coated protein against *Verticillium dahliae*

**DOI:** 10.1371/journal.pone.0349566

**Published:** 2026-05-27

**Authors:** Mahboobeh Nouri, Mojtaba Keykhasaber, Mahdi Pirnia, Mohammad Amin Miri, Shirahmad Sarani, Hoseyn Kamaladini

**Affiliations:** 1 Department of Plant Pathology, Faculty of Agriculture, University of Zabol, Zabol, Iran; 2 Department of Food Science and Technology, Faculty of Agriculture, University of Zabol, Zabol, Iran; 3 Electrospinning Research Laboratory, Central Laboratory, University of Zabol, Zabol, Iran; 4 Department of Biology, Faculty of Sciences, University of Zabol, Zabol, Iran; University of Waterloo, CANADA

## Abstract

*Verticillium dahliae* is a devastating soil-borne fungal pathogen causing wilting in over 400 plant species, leading to significant economic losses. While nucleic acid-based biocontrol strategies like dsRNA-induced consistent with gene-silencing activity show promise, their application is hindered by poor environmental stability and high production costs. Double-stranded oligodeoxynucleotides (oligoDNAs) offer a stable and cost-effective alternative, but require efficient delivery systems. This study aimed to develop and evaluate the efficacy of novel antifungal microcapsules, encapsulating oligoDNAs (targeting the *Clp-1* and *HiC-15* genes of *V. dahliae*) within a whey protein concentrate (WPC) matrix via electrospraying, for controlling Verticillium wilt. The physicochemical properties of the microcapsules were characterized using SEM, TEM, DLS, FTIR, XRD, TGA, DSC, and BET. Antifungal activity was assessed through *in vitro* colony growth assays and fluorescence microscopy to visualize oligoDNA uptake. *In planta* efficacy was evaluated on tomato seedlings by calculating the Disease Index (DI) and Area Under the Disease Progress Curve (AUDPC) over 30 days. Characterization confirmed the successful formation of spherical, oligoDNA-loaded WPC microcapsules with enhanced thermal stability. *In vitro*, the encapsulated oligoDNA (oligoDNA159 + 166) significantly inhibited fungal colony growth compared to controls, while WPC alone showed no effect. Fluorescence microscopy revealed that Cy3-labeled oligoDNA associated with fungal hyphae, producing discrete punctate signals consistent with cellular uptake, though definitive confirmation of intracellular localization requires higher-resolution imaging techniques. In the greenhouse trial, the encapsulated oligoDNA treatment exhibited the strongest suppression, with a mean DI of 50 (± 10.0) on day 30, corresponding to a 47% reduction compared to the pathogen control. This study demonstrates that WPC-encapsulated oligoDNA is an effective, sustainable, and specific biocontrol agent against *V. dahliae*. The dual mode of action—direct inhibition of fungal growth and potential host-induced resistance—positions this technology as a promising, environmentally friendly strategy for managing vascular wilt diseases.

## Introduction

The growing global population and limited natural resources have increased the demand for sustainable agricultural production [1]. However, the outbreak of plant diseases caused by pathogens such as fungi, bacteria, and viruses is considered one of the major challenges in this field [2]. One of the most significant fungal pathogens, responsible for vascular wilt in over 400 plant species worldwide, is *Verticillium dahliae* Kleb. This soil-borne fungus attacks susceptible plants through the roots, damaging their vascular tissues [3,4]. It causes millions of dollars in annual losses worldwide [5].

Identification of resistant cultivars/genotypes to Verticillium is one of the important strategies used in disease management. Some studies have been focused on evaluating resistance to Verticillium and/or detecting resistance gene analogs (RGAs) in cultivars [4,6]. Recent studies have led to the development of novel and low-risk methods for plant disease management. Powder and liquid formulations of potent yeast strains showed high efficacy in attenuating disease severity and toxin production of *Aspergillus flavus* [7]. Essential oils showed great potential for controlling plant pathogens, but due to their volatility, their effectiveness is lost over time [8]. Encapsulation of essential oils in biodegradable, edible protein coatings such as zein, has been shown to increase their efficacy and reduce disease severity [9–11]. Moreover, the use of fungal metabolites for the green synthesis of zinc oxide nanoparticles have also raised hopes for the control of plant pathogens [12].

The development of biological control strategies based on biomolecules such as nucleic acids has emerged as an innovative and environmentally friendly approach for managing plant diseases [13]. Numerous studies have demonstrated that the topical application of dsRNA on plants can effectively prevent the spread of a wide range of plant diseases caused by fungi, oomycetes, and viruses [13,14]. For example, the use of dsRNA has been successful in controlling fungal diseases such as *Fusarium* and *Botrytis* in crop plants [15,16]. The mechanism of action of dsRNA is based on silencing essential pathogen genes, where these molecules interfere with the expression of key genes, thereby inhibiting the growth and proliferation of pathogens [ 1,17].

One of the main limitations in the practical application of dsRNA is its low stability in the environment. Environmental factors such as light, temperature, and degrading enzymes can quickly damage dsRNA and reduce its efficacy [ 1,18,19]. Additionally, its design and synthesis are highly complex and expensive. In contrast, the structure of DNA oligonucleotides is very similar to that of RNA molecules, which are naturally used by cells, making DNA oligos a viable low-cost alternative. Their production and purification are inexpensive and easy, and they are highly stable [20]. The DNA oligonucleotide technology was first successfully used in plant cells to modify the expression of the transcription factor SUSIBA2 [21]. The researchers’ results demonstrated that antisense oligonucleotides were effectively delivered into the leaves and reached the nuclei and chloroplasts [21,22].

To enhance the stability of oligonucleotides when applied to plants, various methods such as encapsulation have been investigated [23]. Nanoencapsulation systems create a protective coating around DNA oligonucleotides, shielding them from degrading factors and enabling their controlled release at the target site [23,24]. Given the sensitivity of bioactive compounds, various encapsulation methods have been developed. The electrospraying process, or microencapsulation using electrohydrodynamic processes, is a simple and effective method for preserving and enhancing the bioavailability of active compounds [25]. In the electrospraying process, various proteins—including whey protein isolate (WPI), soy protein isolate, egg albumin, collagen, gelatin, zein, wheat gluten, and casein—have been investigated and evaluated [ 25,26]. Among these, whey protein concentrate (WPC), as one of the main by-products of the dairy industry, holds particular significance. WPC contains a mixture of proteins, notably beta-lactoglobulin, alpha-lactalbumin, and serum albumin, and is recognized as a rich source of natural emulsifiers [27]. It has been proposed as a suitable candidate for encapsulation via electrohydrodynamic processes due to its excellent electrospray ability and effective performance as a carrier for bioactive compounds [28–31].

Previous research has established that two virulence genes in *V. dahliae* – the Ca² ⁺ -dependent cysteine protease (*Clp-1*) and isotrichodermin C-15 hydroxylase (*HiC-15*) – are critical for fungal pathogenicity. These genes are specifically targeted by miR166 and miR159, respectively, which were identified in resistant cotton cultivars [32]. Building on this finding, the current study investigates the antifungal potential of microencapsulated double-stranded oligoDNAs (corresponding to microRNA166 and microRNA159) produced via electrospraying using whey protein concentrate (WPC), an edible and biodegradable polymer, for controlling *V. dahliae*.

## Materials and methods

### Preparation of WPC solution

WPC solutions (30% w/v) were prepared by dissolving the required amount of WPC in sterile distilled water with gentle stirring at room temperature. The solutions were kept at room temperature for 24 hours, and their pH was adjusted to 6 [33].

### OligoDNA design and preparation of oligo DNA-Loaded WPC microparticles via electrospraying

oligoDNAs were designed based on microRNA159 and microRNA166 sequences. The specific sequences that were synthesized, and purified by Pishgam Biotech Company, Iran, were as follows: oligoDNA 159 (5´-TTTGGATTGAAGGGAGCTCTA-3´)(GC% = 33.3) and oligoDNA 166 (5´-TTCGGACCAGGCTTCATTCCCC-3´) (GC% = 19.1). To encapsulate oligo DNA within WPC microparticles, 20 ng/μL of oligo DNA was added to the WPC solution [13]. The solutions were stirred at room temperature for 30 minutes and then subjected to electrospraying. Microcapsules were prepared using an ES1000 electrospray device (Fanem Co., Iran). A 5 mL syringe was filled with the electrospray solution and mounted on the device. The needle-to-collector distance was set to 14 cm. oligo DNA-loaded WPC microcapsules were electrosprayed at 18 kV with a flow rate of 0.5 mL/h for 10 hours at room temperature [33].

### Scanning electron microscopy (SEM)

The morphology of microcapsules was analyzed using a scanning electron microscope (EM-8000F, KYKY, Germany) after coating with a gold-palladium mixture (20 nm thickness) via sputter coating. The average diameter of electrosprayed microcapsules was determined by randomly measuring 100 microcapsules from each SEM image using ImageJ software, with an accelerating voltage of 20 kV [34].

### Transmission electron microscopy (TEM)

To confirm encapsulation and the presence of oligoDNA, samples were analyzed using transmission electron microscopy (TEM) with negative staining. In this technique, samples are coated with a high electron-density material (such as uranyl acetate or phosphotungstic acid). This staining agent penetrates the surrounding sample space, causing areas with lower electron density (such as microcapsules) to appear as brighter regions against a dark background. Consequently, the white and dark spots in TEM images represent differences in the sample’s electron density [35]. In this study the morphology of microcapsules and oligo DNA loading efficiency were evaluated using a Philips EM208S 100KV TEM (Netherlands).

### Particle size distribution and hydrodynamic diameter

The average hydrodynamic diameter (dls) and particle size distribution of freshly diluted samples were determined using dynamic light scattering (DLS) with a Zetasizer Nano ZS instrument (Malvern Instruments, Worcestershire, UK) [36].

### Fourier transform infrared (FTIR) spectroscopy

To study the chemical structure of WPC microcapsules loaded with oligoDNA, FTIR analysis was performed [30]. Polypeptide and protein repeating units exhibit specific IR absorption bands for amides A and B, as well as amides I to VII. Among these, amide I (C = O stretching vibration at 1580–1720 cm ⁻ ¹) and amide II (N–H bending vibration at 1450–1600 cm ⁻ ¹) are the two key vibrational bands of the protein backbone. However, changes in secondary structure are more clearly observed in the amide I absorption bands due to the minor contributions of C–N stretching, C–C–N deformation, and in-plane N–H bending [37–39]. All measurements were carried out using a Thermo Nicolet spectrophotometer (AVATAR 370 FTIR, USA) in the range of 4000–400 cm ⁻ ¹ with a resolution of 4 cm ⁻ ¹ [40].

### X-ray diffraction (XRD) analysis

X-ray diffraction was employed to assess the mixing and dispersion of different compounds and to determine the physical state of oligoDNA within the electrosprayed whey protein microcapsules. The samples were scanned using an XRD analyzer (Unisantis, XMD-300) with CuKα radiation (λ = 1.5418 Å) at an incident angle (2θ) ranging from 5° to 40° at room temperature. The X-ray diffraction spectra were evaluated comparatively [41].

### Differential scanning calorimetry (DSC)

DSC measurements were performed using a Perkin Elmer STA6000 differential scanning calorimeter at a heating rate of 10°C/min. Samples weighing 5 mg were heated under a nitrogen (N₂) atmosphere from 25°C to 400°C [34].

### Thermogravimetric analysis (TGA)

TGA provides essential information on thermal stability, decomposition temperature, and residual mass of a sample by measuring weight changes as a function of temperature. Differential scanning calorimetry (DSC) is another powerful tool that offers insights into thermal transition events by measuring the heat flow associated with these processes. In this study, simultaneous thermal events were measured using both TGA and DSC techniques. Additionally, derivative thermogravimetry (DTG) was employed as a function of applied temperature to provide further data and more precise insights into thermal decomposition processes. To evaluate the thermal stability of the microcapsules, a Perkin Elmer STA6000 instrument was used. Approximately 5 mg of the sample was placed in a platinum crucible and heated under a nitrogen flow of 40 mL/min at a heating rate of 10°C/min from 25°C to 750°C.

### Pore size and volume measurement (BET)

The pore size analysis of the microcapsules was performed using Brunauer-Emmett-Teller (BET) and Barrett-Joyner-Halenda (BJH) methods based on nitrogen (N₂) adsorption-desorption isotherms. A BELSORP-mini II instrument was used for this analysis. This model is employed to determine the specific surface area of porous and powdered materials using the slope and intercept of the BET linear plot, where a steeper slope corresponds to a higher surface area [42]. In this analysis the nitrogen adsorption-desorption isotherm provides information about the surface area, pore volume, and pore size distribution of the sample. The difference between the adsorption (ADS) and desorption (DES) curves indicates the presence of hysteresis, which is characteristic of a mesoporous structure [43,44].

### Fluorescence microscopy

**Fungal material and culture conditions:** *Verticillium* spp. isolates were maintained on potato dextrose agar (PDA; Difco) and subcultured onto fresh plates 5–7 days prior to experimentation. For liquid culture assays, mycelial plugs (5 mm) from actively growing colonies were transferred into potato dextrose broth (PDB) and incubated at 22–24 °C with shaking at 120 rpm for 48 h to obtain young hyphae suitable for uptake assays. Growth conditions were selected to ensure hyphal vitality and endocytic activity, following previous work on nucleic acid uptake in *Verticillium* and other filamentous fungi [45,46].

**Fluorescent labeling:** For visualization in fungal uptake studies, single-stranded DNA oligonucleotides (21–25 nt) were synthesized with a 5′-Cy3 fluorophore (Integrated DNA Technologies). Each oligo was resuspended in nuclease-free water and diluted to a working concentration of 100 nM. All fluorescent labeling and handling procedures were conducted according to established protocols for dsRNA/ssRNA fluorescent tagging in fungal systems [46,47].

**Oligo treatment of fungal hyphae:** Fungal cultures were collected by gentle centrifugation (3000 × g, 5 min) and washed twice with sterile phosphate-buffered saline (PBS). Approximately 200 µL of hyphal suspension was transferred to sterile microscopy chamber slides. Cy3-labeled oligoDNA was added directly to the chamber to achieve the desired final concentration, and samples were incubated for 1–3 h at room temperature in the dark. Incubation time and concentrations were selected based on established fungal RNA uptake assays demonstrating time-dependent association consistent with uptake via endocytosis [45,48]. Untreated control samples received an equivalent volume of PBS without Cy3-labeled oligo.

**Microscopy and imaging parameters:** Fluorescence imaging was performed using an epifluorescence microscope equipped with 10× and 40 × objectives (Plan Fluor series) and a Cy3-compatible filter set (excitation 525–560 nm; emission 570–620 nm). Exposure times (150–500 ms) were kept constant across treatments and controls to ensure comparability. Images were captured with a high-sensitivity CMOS camera.

At 10 × magnification, wide-field imaging was used to assess general fluorescence distribution across fungal biomass. At 40 × magnification, higher-resolution imaging enabled identification of discrete fluorescent puncta and association with hyphal structures, following imaging strategies commonly used in fungal RNA uptake analyses [45,46].

**Image processing and analysis:** Raw images were processed using FIJI/ImageJ. Background subtraction (rolling ball radius 50 pixels) was applied uniformly across all samples. Fluorescence detected in hyphae exposed to Cy3-oligo was compared to untreated controls to confirm that observed signals were not attributable to intrinsic autofluorescence, consistent with previous studies on fluorescent nucleic acid uptake in fungi [46,49].

### Analysis of gene expression

**Fungal culture conditions:** An isolate of *Verticillium dahliae* race 1 (isolate No. Iran224c) was used in this study. The isolate, originally obtained from tomato, was provided by the Iranian Research Institute of Plant Protection in Tehran, Iran, where its pathogenicity was confirmed. The fungus was cultured and maintained on potato dextrose agar (PDA). For experimental setup, fresh subcultures were prepared 5–7 days in advance. To generate uniform mycelial biomass, actively growing hyphal tips were transferred to Czapek Dox liquid medium and incubated for 7 days at room temperature with constant agitation (100 rpm).

**OligoDNA treatment application:** Synthetic oligoDNA constructs targeting the *Clp-1* and *HiC-15* genes were encapsulated within a whey protein concentrate (WPC) matrix. To verify the suppression of the target genes by these oligonucleotides, liquid fungal cultures were treated with both the encapsulated formulations and non-encapsulated oligoDNAs at a concentration of 20 ng/µL. The treated mycelial suspensions were subsequently plated onto potato dextrose agar (PDA) and incubated under standard conditions (25 °C). An untreated culture, which received no oligoDNAs, served as the negative control.

**RNA extraction and cDNA synthesis:** Total RNA was extracted from 4-day-old control and treated hyphae using the Zand Biotech RNA extraction kit (Zand Biotechnology Company, Iran) according to the manufacturer’s protocol. The treatments included control untreated hyphae (P), hyphae treated with encapsulated and non-capsulated oligoDNA159 and oligoDNA166. RNA purity and integrity were assessed via spectrophotometry (Unico USA UV-2100) and agarose gel electrophoresis. Residual genomic DNA was eliminated by DNase I treatment. First-strand cDNA was synthesized from 1 µg of total RNA using a reverse transcription kit with oligo(dT) primers. Prior to quantitative PCR analysis, the resulting cDNA samples were diluted to a uniform working concentration.

**Quantitative real-time PCR (qPCR):** Gene expression levels of *Clp-1* and *HiC-15* were quantified using SYBR Green-based RT-qPCR on a Rotor-Gene 6000 real-time thermal cycler (Corbett Research). Each 20 µL reaction mixture contained 10 µL of SYBR Green Master Mix (Ampliqon Company, Denmark), 0.4 µM of each gene-specific primer, and 2 µL of the diluted cDNA template. The thermal cycling protocol included an initial denaturation at 95 °C for 3 minutes, followed by 40 cycles of 95 °C for 10 seconds and 60 °C for 30 seconds. A melt curve analysis was performed post-amplification to confirm the specificity of the PCR products. The experiment included three biological replicates for each treatment, each run with two technical replicates.

**Statistical analysis:** To meet the assumptions of parametric testing and stabilize variance, the mean Ct values for each sample were transformed using the 2^^–ΔΔCt^ method prior to statistical analysis, with the fungal *GAPDH* gene serving as a stable internal reference for normalization. Normality and homogeneity of variance were verified using Shapiro–Wilk and Levene’s tests, respectively. A one-way ANOVA (or Welch’s ANOVA when variance heterogeneity was suspected) was used to assess overall treatment effects. All analyses were performed using IBM SPSS Statistics 27 with significance set at p < 0.05.

### Colony growth

A spore suspension of *Verticillium* was prepared at a concentration of 1 × 10⁶ spores/mL. The experimental treatments consisted of empty WPC microcapsules, as well as encapsulated and non-encapsulated oligoDNAs, each dissolved in 10 µL of the fungal spore suspension to achieve a final concentration of 20 ng/µL. An untreated spore suspension served as the control (P). After 30 minutes of incubation, each mixture was cultured at the center of a 90 mm Petri dish containing potato dextrose agar (PDA) [13]. For each of five biological replicates per treatment, colony diameters were measured on day 12 post-culturing using ImageJ software, and mean colony areas were calculated for each experimental group. Growth rates were statistically compared using a one-way analysis of variance (ANOVA), with significance set at p < 0.05.

### Assessment of encapsulated oligoDNA efficacy on disease progress

**Plant material and growth conditions:** Tomato seedlings (*Solanum lycopersicum* L.) of the Verticillium wilt-susceptible ‘Falat’ cultivar at the two- to four-leaf stage were used. Plants were grown in a soil mixture of cocopeat, perlite, and peat moss (1:2:2 v/v/v), which was sterilized by autoclaving at 121°C for 60 minutes on two consecutive days prior to use [50,51]. Each experimental unit consisted of a single seedling planted in a plastic pot (15 cm diameter × 15 cm height).

**Pathogen inoculation and treatment application:** A spore suspension of *Verticillium* sp. was prepared at a concentration of 10⁶ spores/mL. Encapsulated oligoDNA samples were mixed into the spore suspension at a final concentration of 20 ng/µL, following the method described by Qiao et al. [13]. For inoculation, seedlings were carefully removed from the soil, their roots were washed under running water, and then submerged in the respective treatment suspension for 5 minutes [52]. Following inoculation, the seedlings were transplanted into prepared pots and placed in a growth chamber. The chamber was maintained at a constant 25°C with a 12-hour photoperiod and a relative humidity of 60–70%. A total of five treatment groups were included in the experiment: a negative control receiving only water (no pathogen, no oligoDNA); a pathogen-positive control inoculated with spore suspension only; a vehicle control treated with spore suspension containing the WPC encapsulant alone; and two oligoDNA treatment groups receiving spore suspension containing an equal combination of oligoDNA159 + oligoDNA166, either in encapsulated or non-encapsulated form.

**Experimental design and disease assessment:** The experiment was arranged in a completely randomized design with ten replicate plants per treatment. Disease severity was assessed every three days for one month using a 0–5 visual rating scale adapted from Abada et al. [53], based on foliar symptoms of wilting and chlorosis; 0: No symptoms, 1: Mild leaf wilting, 2: Yellowing of some leaves, 3: Moderate wilting and significant yellowing, 4: Severe wilting and stunting, 5: Plant death. The primary symptoms considered for rating were wilting, leaf yellowing (chlorosis), and varying degrees of dwarfing.

**Data analysis:** The Disease Index (DI) was calculated for each plant using the following formula


$$\begin{array}{c} \text{DI}=\;\frac{\sum (i\times p_{i})}{\text{max}\times p_{total}}\times \text{1}\text{0}\text{0} \end{array}$$

i = Disease severity rate (rating score, from 0 to 5). Pi = Number of plants that achieved severity rate i imax = Highest disease rate on the scale (equal to 5). P total = Total number of inoculated plants.

Additionally, the area under the disease progress curve (AUDPC) was determined using the following formula [54]:


$$\begin{array}{c} \text{AUDPC }=\;\sum_{i=\text{1}}^{n-\text{1}}\left(\frac{y_{i}+y_{i+\text{1}}}{\text{2}}\right)(t_{i+\text{1}}-t_{i}) \end{array}$$

One-way ANOVA analyses with significance set at p < 0.05 were conducted to compare the mean disease progress results.

## Results

### Morphology and diameter determination by SEM

**Fig 1:**
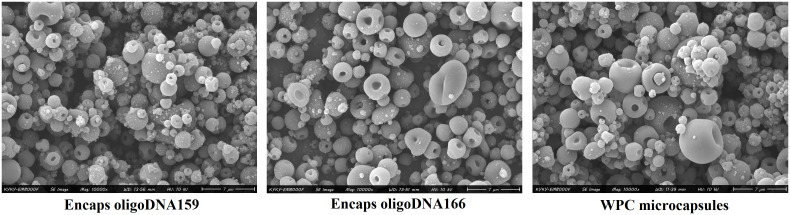
SEM images of electrosprayed structures. Images illustrate encapsulated oligoDNA159 and oligoDNA166, and WPC capsules with 10% concentration and 10000 magnification.

SEM images of the electrosprayed structures for WPC, and WPC loaded with oligoDNA159 and oligoDNA166 are presented in Fig 1. The images reveal that in all treatments, spherical microcapsules with smooth surfaces were successfully obtained via electrospraying from an aqueous solution containing 30% (w/w) protein concentration. The mean diameters of WPC microcapsules without oligonucleotides, WPC microcapsules containing oligoDNA159, and WPC microcapsules containing oligoDNA166 were 0.20831 µm, 0.35886 µm, and 0.26744 µm, respectively.

### Transmission electron microscopy (TEM) analysis

**Fig 2:**
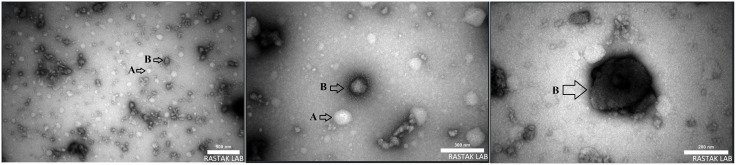
TEM images of WPC microcapsules with and without oligoDNA. The images reveal that WPC particles without oligoDNA (A) appear as brighter spots against the dark spots that represent encapsulated oligoDNAs **(B)**.

TEM analysis (Fig 2) showed that WPC microcapsules without oligoDNA appeared as bright spherical or near-spherical particles (≈50–200 nm), with noticeable size heterogeneity and some aggregation, consistent with DLS findings. The absence of uniform surface layers indicated that no active material was present.WPC microcapsules containing oligoDNA displayed a similar spherical morphology. Due to the low electron density of oligoDNA (21 nucleotides), DNA-loaded regions were not directly visible in TEM images. However, the presence of larger aggregates and increased particle size suggested strong interactions between whey proteins and oligoDNA, supporting successful encapsulation. Some particles showed uniform surface layers, providing morphological evidence of oligoDNA incorporation. Overall, TEM observations confirmed the formation and structural characteristics of both unloaded and oligoDNA-loaded WPC microcapsules and aligned with DLS results.

### Particle size and polydispersity index (PDI)

The particle size and polydispersity index (PDI) values for the micro-carrier systems are presented in Table 1. PDI, a key indicator of dispersion homogeneity, showed values of 0.69 for pure WPC, 0.52 for WPC + oligoDNA159, and 1.00 for WPC + oligoDNA166, indicating that all formulations contained heterogeneous particle populations.

**Table 1:** The mean particle size and polydispersity index of the samples.

Sample	Polydispersity Index (PDI)	Hydrodynamic Diameter (nm)
**WPC**	0.690	434.2
**MC-WPC oligoDNA159**	0.522	754.4
**MC-WPC oligoDNA166**	1.000	2075

**Fig 3:**
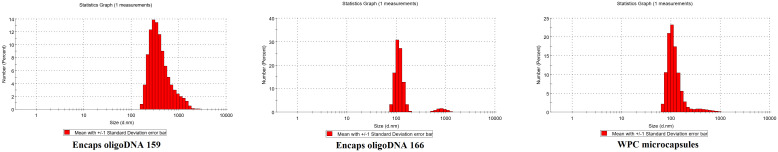
The particle size distribution histogram (number-based) obtained by dynamic light scattering. All three graphs demonstrate an asymmetric distribution with the majority of particles concentrated in the 100-1000 nm range.

Number-based DLS particle size distributions (Fig 3) revealed asymmetric histograms with most particles falling within the 100–1000 nm range. Particle counts decreased steadily with increasing size, suggesting that large aggregates were present but numerically scarce. The broad standard deviations observed across samples confirmed size heterogeneity and aligned with the high PDI values. Despite the variability, the presence of predominantly small particles with a limited fraction of larger aggregates suggests that the formulations remain suitable for controlled-release applications, where gradual diffusion from heterogeneous carrier populations can be advantageous.

### Fourier transform infrared spectroscopy (FTIR)

FTIR analysis was used to evaluate the amide bands of WPC and to determine how oligoDNA incorporation affected the molecular structure of the microcapsules. In nucleic acids, the 1600–1800 cm ⁻ ¹ region typically corresponds to C = O stretching in the carbonyl groups of nitrogenous bases, and strong peaks in this region confirm the presence of DNA.

Pure WPC exhibited characteristic absorption bands, including N–H stretching at 3433.11 cm ⁻ ¹, aliphatic C–H stretching at 2925.86 and 2862.21 cm ⁻ ¹, Amide I at 1643.26 cm ⁻ ¹, Amide II at 1525.61 cm ⁻ ¹, and aromatic C = C bending at 1461.96 cm ⁻ ¹. For oligoDNA159, diagnostic bands appeared at 3458.19, 2075.30, and 1637.48 cm ⁻ ¹, while oligoDNA166 showed peaks at 3477.47 and 1650.98 cm ⁻ ¹, with the latter two reflecting DNA C = O stretching vibrations.

**Fig 4:**
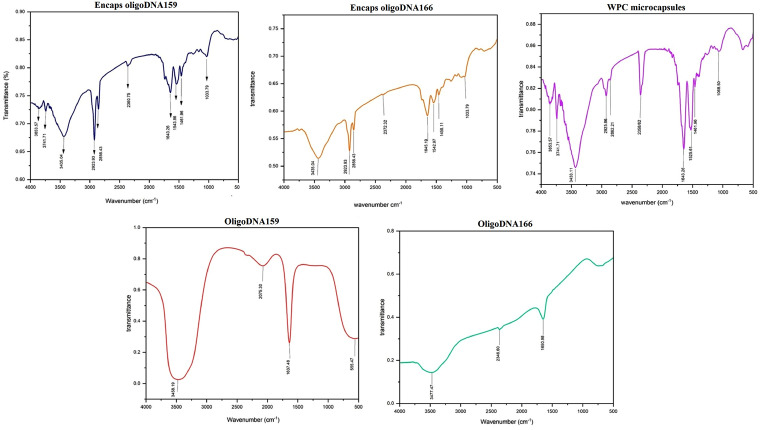
The FTIR spectral bands. Images show the FTIR spectral bands of oligoDNA159, oligoDNA166, WPC microcapsules, and encapsulated oligoDNA159 and oligoDNA166.

Fig 4 compares the FTIR spectra of pure oligoDNAs, WPC, and WPC microcapsules loaded with oligoDNA. While no new functional groups emerged after encapsulation, shifts in peak position and changes in band intensity were evident. The Amide I band remained unchanged in microcapsules containing oligoDNA159 but shifted to a higher wavenumber for microcapsules containing oligoDNA166. Likewise, the Amide II band shifted upward for both oligoDNA-loaded microcapsules, indicating structural alterations in whey protein during encapsulation. Some DNA-related peaks were absent or masked due to the low concentration of oligoDNA or overlap with strong WPC signals. Differences between the spectra of oligoDNA159 and oligoDNA166 were also observed, likely resulting from variations in their nucleotide compositions. These results collectively indicate that oligoDNA is physically entrapped, rather than chemically bonded, within the WPC microcapsules.

### X-ray diffraction (XRD) analysis

**Fig 5:**
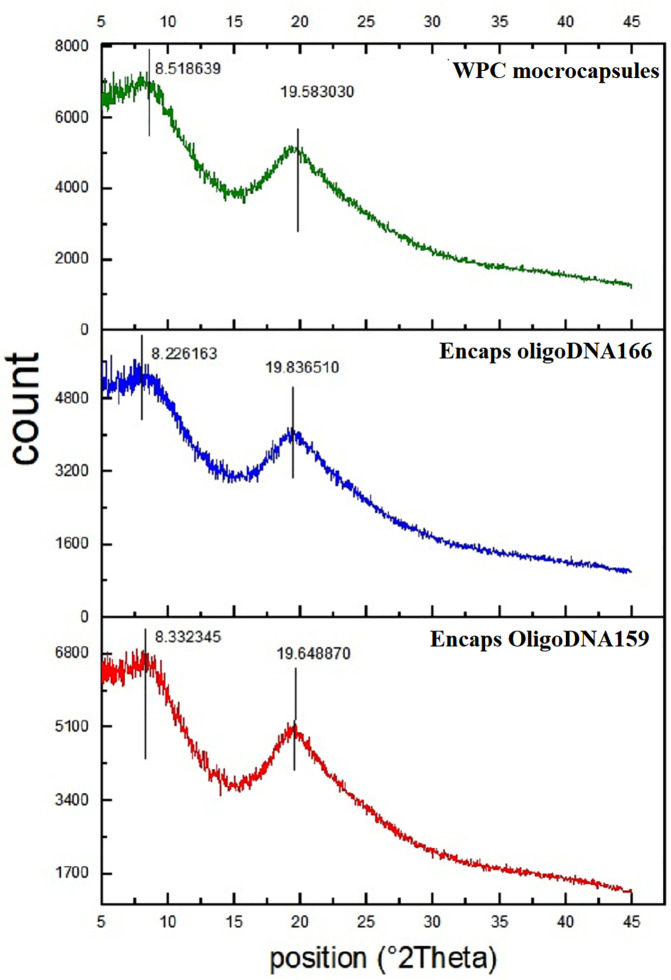
The XRD patterns. This image represents the XRD patterns of WPC microcapsules, and Encapsulated oligoDNA159 and oligoDNA166.

X-ray diffraction patterns are used to examine the crystalline structure of biopolymeric materials. Fig 5 shows the XRD patterns of WPC microcapsules, and Encapsulated oligoDNA159 and oligoDNA166. Two distinct peaks were observed in the pure WPC diffractogram at 8.51° 2θ (d-spacing = 10.3 Å) and 19.58° 2θ (d-spacing = 4.52 Å). The first peak is attributed to the interhelical packing distance, while the second peak corresponds to the d-spacing of α-helical structures. In this study, the microcapsule structure appears predominantly amorphous, with possible small nanocrystalline regions, as no sharp and well-defined peaks indicative of high crystallinity were detected. The first diffraction peaks of oligoDNA159 and oligoDNA166 in WPC were observed at 8.33° 2θ (interlayer spacing of 10.6 Å) and 8.22° 2θ (interlayer spacing of 10.7 Å), respectively, while the second diffraction peaks appeared at 19.64° 2θ (interlayer spacing of 4.51 Å) and 19.83° 2θ (interlayer spacing of 4.4 Å), respectively. The peak intensities for oligoDNA159 remained nearly unchanged, whereas those for oligoDNA166 decreased. Additionally, the first peak (2θ = 8.51°) shifted to a lower angle in both treatments, and the second peak (2θ = 19.58°) shifted to a higher angle in both treatments.

### Thermogravimetric analysis (TGA)

**Fig 6:**
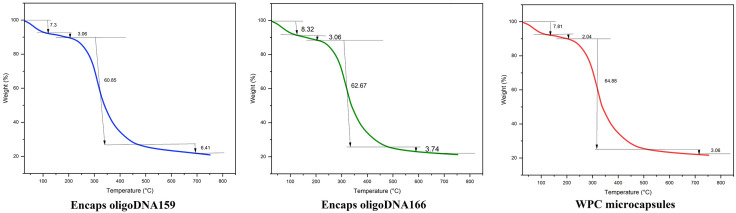
Thermogravimetric analysis (TGA). Illustrates the TGA behavior of WPC microcapsules, and Encapsulated oligoDNA159 and oligoDNA166.

Fig 6 presents the thermal decomposition profiles of WPC, WPC–oligoDNA159, and WPC–oligoDNA166 microcapsules. All samples exhibit similar TGA and DSC patterns, consisting of four distinct degradation stages:

1. Moisture Loss (30–150 °C): Initial weight reductions of 7.3%, 8.32%, and 7.81% correspond to evaporation of free and surface-bound water.
2. Solvent and Bound Water Removal (150–250 °C): A second mass loss of 3.06%, 3.06%, and 2.04% is associated with the release of bound water within the protein matrix and residual processing solvents.
3. Major Structural Degradation (250–400 °C): The dominant weight loss—60.85%, 62.67%, and 64.88%—reflects thermal denaturation and decomposition of whey proteins (notably β-lactoglobulin and α-lactalbumin), disruption of disulfide linkages, and degradation of encapsulated oligonucleotides.
4. Decomposition of Resistant Residues (400–800 °C): Final losses of 6.41%, 3.74%, and 3.06% indicate breakdown of thermally resistant organic structures and carbonaceous residues.

Approximately 22% residue remained at 800 °C for all samples, attributed to inorganic minerals and highly stable carbon structures.

Overall, microcapsules containing oligonucleotides exhibited slightly reduced total mass loss (60–62%) compared with WPC alone (~64%), suggesting enhanced thermal stability. This increased stability likely results from molecular interactions between whey proteins and the oligonucleotides, rather than differences in moisture content, which was similar among all formulations.

### Differential scanning calorimetry (DSC)

**Fig 7:**
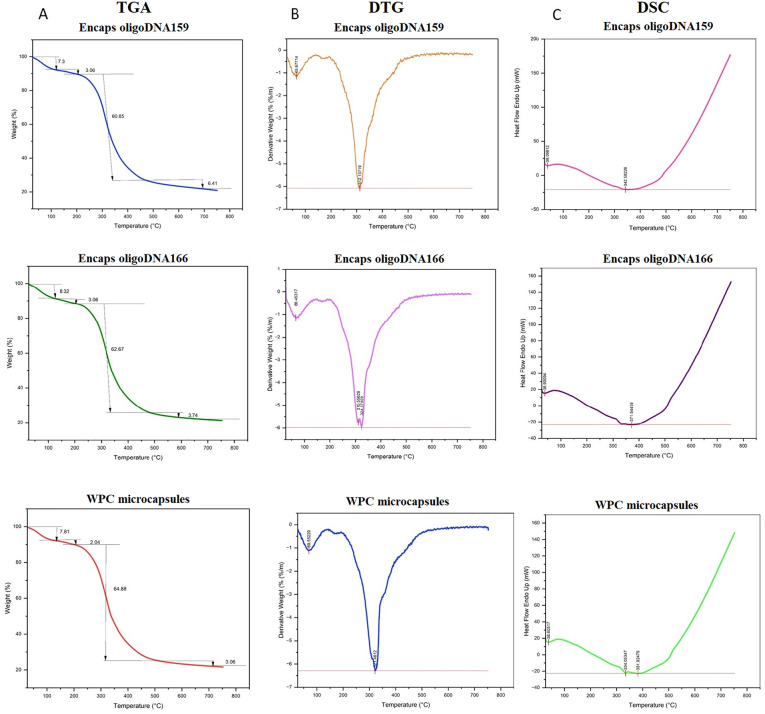
Differential scanning calorimetry (DSC). The TGA **(A)**, DTG **(B)**, and DSC (C) analysis curves for WPC microcapsules, and Encapsulated oligoDNA159 and oligoDNA166.

DSC provides insights into thermal transition events by measuring the heat flow associated with these events in the sample. Fig 7 shows the DSC analysis curves for WPC, WPC-oligoDNA159, and WPC-oligoDNA166 microcapsules. The temperature range of 30–150°C, where a small endothermic peak is observed at around 39°C. This peak indicates energy absorption for the evaporation of surface moisture and free water from the microcapsules. This finding aligns with the initial weight loss in the TGA curve within the same temperature range. The temperature range of 150–300°C, where the curve shifts downward (endothermic), indicating the onset of phase transitions in the protein structure.

A major endothermic valley is observed at 342.8°C, 371.9°C, and 381.9°C, respectively, indicating significant endothermic processes. These include thermal denaturation of whey proteins, cleavage of chemical bonds within the protein structure, and thermal degradation of oligonucleotides. This endothermic valley closely aligns with the major weight-loss stage in the TGA analysis (observed in the 250–400°C range). A sharp increase in heat flow occurs at temperatures above 400°C, where the curve shifts continuously upward (exothermic). This trend suggests more complex thermal processes, and likely final decomposition of residual materials. This behaviour correlates with the final weight-loss stage in the TGA analysis.

### BET (Brunauer–Emmett–Teller) analysis

The specific surface areas of the microcapsules, calculated using the BET method, are presented in Table 2. Pure WPC microcapsules exhibited a specific surface area of 4.83 m²/g, which is considered low to moderate for particulate materials. WPC microcapsules loaded with oligoDNA159 showed a reduced surface area of 0.55 m²/g, while those loaded with oligoDNA166 demonstrated an intermediate value of 2.97 m²/g.

**Table 2:** Presents the specific surface area for WPC mocrocapsules, and encapsulated oligoDNA159 and oligoDNA166 that was calculated using the slope and intercept of the BET linear.

Parameter	WPC	Encapsulated oligoDNA159	Encapsulated oligoDNA166
**BET Surface Area (m²/g)**	4.83	0.55	2.97
**BJH Pore Volume (cm³/g)**	0.0079	0.0014	0.0048
**Dominant Pore Size (nm)**	1.6	1.2	1.9

**Fig 8:**
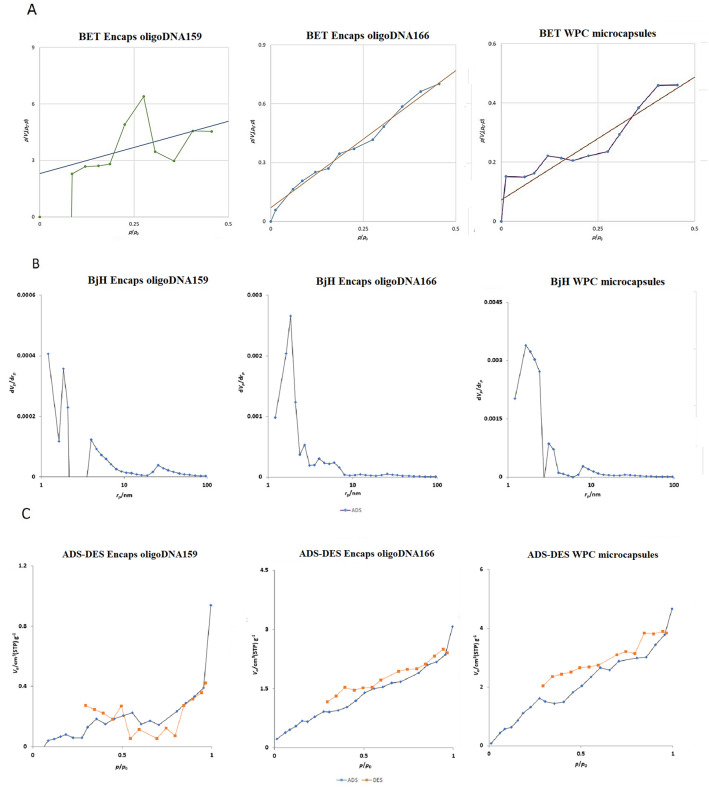
Pore volume and size distribution. Images showe the BET **(A)**, BJH **(B)**, and ADS (C) analysis curves for WPC mocrocapsules, and encapsulated oligoDNA159 and oligoDNA166.

The Barrett-Joyner-Halenda (BJH) model was employed to evaluate pore volume and size distribution (Fig 8). All samples exhibited a dominant peak in the 1–3 nm range, indicating the presence of micropores (<2 nm) and small mesopores (2–20 nm). The decline in pore volume beyond 20 nm confirms the absence of macropores (>50 nm), indicating a predominantly micro-mesoporous structure.

Pore volume measurements revealed significant differences among the samples (Table 2). Pure WPC microcapsules exhibited a pore volume of 0.0079 cm³/g. This decreased by approximately 82% to 0.0014 cm³/g in WPC microcapsules containing oligoDNA159, and by approximately 40% to 0.0047 cm³/g in those containing oligoDNA166. This substantial decrease following oligoDNA incorporation suggests pore blockage by oligonucleotide molecules and structural modifications within the protein matrix. The more pronounced effect observed with oligoDNA159 may indicate sequence-dependent interactions between the oligonucleotides and whey protein components.

All three samples exhibited Type IV adsorption isotherms with H3-type hysteresis loops (according to IUPAC classification), consistent with the presence of mesoporous structures and slit-shaped pores formed by aggregates of plate-like particles.

### Analysis of fluorescence microscopy image of *Verticillium* spp. treated with Cy3-labeled oligo-DNA

Fluorescence microscopy of *Verticillium* spp. exposed to Cy3-labeled oligo-DNA demonstrated clear evidence of fluorophore-associated signals at both 10× and 40 × magnifications (S1 Fig). At 10 × , strong localized fluorescence was detected as dense clusters of bright yellow-red puncta, indicating regions of concentrated oligo interaction within the fungal biomass. At 40 × , the fluorescence pattern resolved into numerous discrete punctate signals distributed along and around hyphal structures, accompanied by weaker diffuse background fluorescence aligned with hyphal filaments. No comparable signal was observed in untreated controls (not shown), confirming that the observed fluorescence originated from Cy3-oligo exposure rather than intrinsic fungal autofluorescence. The presence of discrete fluorescent puncta at higher magnification suggests that the interaction may extend beyond simple surface coating, potentially indicating association consistent with uptake or localized accumulation in fungal structures.

### Encapsulated OligoDNA directly inhibits *Verticillium* colony growth *in vitro*

To determine if the disease-suppressive effects observed *in planta* were due to a direct antifungal activity, we investigated the impact of encapsulated oligoDNA on the *Verticillium* colony growth *in vitro*. The colony growth assay revealed a clear and significant inhibitory effect of the encapsulated oligoDNA treatment on fungal radial expansion. The control treatment (P), consisting of spores alone, exhibited robust and continuous colony growth over the 12-day monitoring period. Similarly, spores mixed with pure WPC microcapsules and non-encapsulated oligoDNAs159 + 166 showed a growth pattern indistinguishable from the control, indicating that the encapsulant material itself had no inherent antifungal properties. In contrast, the treatment where spores were mixed with microcapsules containing oligoDNAs159 + 166 showed a marked suppression of mycelial growth. Quantitative image analysis using ImageJ software confirmed these visual observations.

The effect of treatments on *Verticillium* growth was assessed by measuring fungal colony area (cm²). A one-way ANOVA revealed a significant difference among treatment groups (F(3, 16) = 11.98, p < 0.001) (S1 File, S2 Fig). Due to unequal variances (Levene’s test, p = 0.008), the Welch test confirmed these differences (Welch’s F(3, 8.60) = 17.65, p = 0.001), and post-hoc comparisons were conducted using the Games-Howell test. The pathogen-only control exhibited the largest mean colony area (8.78 ± 1.06 cm²), representing uninhibited fungal growth. The encapsulated oligoDNAs 159 + 166 resulted in the smallest colony area (4.47 ± 0.71 cm², p = 0.001), corresponding to a 49% reduction. The WPC (7.54 ± 1.92 cm²) and non-encapsulated oligoDNAs 159 + 166 (6.83 ± 0.63 cm², p = 0.006) treatments were not significantly different from the pathogen control (p = 0.611). These results demonstrate that both oligoDNA formulations significantly inhibit *Verticillium* growth *in vitro*, with the encapsulated formulation exhibiting the strongest suppressive effect.

### Differential expression of *Clp-1* and *HiC-15* genes in encapsulated, non-encapsulated, and pathogen-exposed states

**Fig 9:**
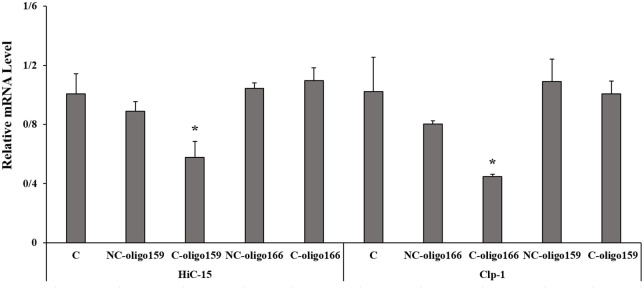
Relative expression levels of *Clp-1* and *HiC-15* genes in fungal hyphae following treatment with oligoDNAs. Gene expression levels of *HiC-15* and *Clp-1* were measured by qRT-PCR and normalized to an internal control (*GAPDH*). Fungal hyphae were treated with non-encapsulated oligo159 (NC-oligo159), encapsulated oligo159 (C-oligo159), non-encapsulated oligo166 (NC-oligo166), or encapsulated oligo166 (C-oligo166). Untreated plants (C) served as the control. Values represent mean relative mRNA levels ± SD (n = 3). Asterisks indicate statistically significant differences compared to the untreated control for each respective gene (p < 0.05; one-way ANOVA followed by appropriate post-hoc test).

One-way ANOVA was performed to evaluate the suppressive effects of encapsulated and non-encapsulated oligonucleotides on *HiC-15* and *Clp-1* gene expression in fungal hyphae (Fig 9). For *HiC-15*, analysis revealed a statistically significant difference among the five experimental groups (F(4,11) = 13.830, p < 0.001), with encapsulated oligoDNA159 exhibiting the strongest suppression of gene expression (0.578 ± 0.107), For *Clp-1*, a significant difference was also observed among the groups (F(4,11) = 10.393, p = 0.001), with encapsulated oligoDNA166 demonstrating the most potent suppression (0.447 ± 0.017). Post-hoc analysis demonstrated that encapsulation significantly enhanced gene suppression for both oligonucleotide types. Encapsulated oligoDNA159 resulted in significantly greater suppression of *HiC-15* expression compared to its non-encapsulated counterpart (mean difference = −0.311, p < 0.05), while encapsulated oligoDNA166 achieved significantly stronger suppression of *Clp-1* expression relative to its non-encapsulated form (mean difference = −0.355, p < 0.05). For both genes, the encapsulated forms consistently exhibited the lowest expression levels, indicating that encapsulation potentiates the gene-silencing activity of these oligonucleotides.

Notably, encapsulated oligoDNA159 achieved significantly greater suppression compared to encapsulated oligoDNA166 (mean difference = −0.519, p < 0.001), non-encapsulated oligoDNA166 (mean difference = −0.467, p = 0.001), and the control group (mean difference = −0.429, p = 0.001) for *HiC-15* expression. Similarly, encapsulated oligoDNA166 demonstrated significantly stronger suppression than all other groups for *Clp-1* expression, including encapsulated oligoDNA159 (mean difference = −0.561, p = 0.004), non-encapsulated oligoDNA159 (mean difference = −0.645, p = 0.001), and the control group (mean difference = −0.575, p = 0.002). These findings indicate differential silencing efficacy between the two oligonucleotide types.

No significant differences in suppression were observed between non-encapsulated oligoDNA159 and encapsulated oligoDNA166, non-encapsulated oligoDNA166, or the control group for *HiC-15* expression, nor between non-encapsulated oligoDNA166 and encapsulated oligoDNA159, non-encapsulated oligoDNA159, or the control group for *Clp-1* expression. Additionally, no significant differences in silencing activity were detected between encapsulated oligoDNA159 and its non-encapsulated counterpart for *Clp-1*, nor between encapsulated oligoDNA166 and its non-encapsulated form for *HiC-15*.

Collectively, these results demonstrate that encapsulated oligoDNA159 achieves significantly stronger suppression of *HiC-15* expression relative to its non-encapsulated form, while encapsulated oligoDNA166 exhibits significantly more potent suppression of *Clp-1* expression compared to its non-encapsulated control. The consistent pattern of encapsulated forms showing the lowest expression levels across both genes confirms that encapsulation enhances the gene-silencing efficacy of these oligonucleotides. However, the significant differences in baseline suppression between encapsulated oligoDNA159 and encapsulated oligoDNA166 indicate divergent intrinsic silencing activities between the two oligonucleotide types, which warrant consideration when evaluating their relative therapeutic potential.

### Encapsulated OligoDNA treatment significantly suppresses verticillium wilt symptoms

To evaluate the antifungal efficacy of encapsulated oligoDNAs against *V. dahliae*, disease severity was assessed in tomato seedlings over a 30-day period using a 0–5 visual rating scale. The Disease Index (DI) was calculated for each treatment group at nine time points (days 6, 9, 12, 15, 18, 21, 24, 27, and 30 post-inoculation) (S2 File, S3 Fig). Table 3 shows the mean Disease Index (± SD) for all treatments over time. Negative controls remained symptom-free (mean = 0). Pathogen-inoculated plants developed severe wilting, reaching a mean DI of 94 (± 8.9) by day 30. WPC treatment alone showed no protective effect (DI = 82 ± 17.9). Non-encapsulated oligoDNAs moderately reduced disease severity (DI = 72 ± 13.0). Importantly, the encapsulated oligoDNA treatment exhibited the strongest suppression, with a mean DI of 50 (± 10.0) on day 30, corresponding to a 47% reduction compared to the pathogen control. These descriptive findings suggest that WPC-encapsulated oligoDNAs provide effective and sustained protection against Verticillium wilt.

**Table 3:** Mean and standard deviation of disease index (DI) in different treatments over time (days 6 to 30 post-inoculation).

	Treatment	Control	Pathogen	Encaps oligos	Non-encaps oligos	WPC	Total
D6	Mean	0/00	34/00	8/00	20/00	52/00	22/80
	Std. De.	0/00	21/91	8/37	18/71	35/64	26/85
D9	Mean	0/00	44/00	10/00	26/00	54/00	26/80
	Std. De.	0/00	20/74	7/07	18/17	33/62	27/34
D12	Mean	0/00	66/00	16/00	32/00	62/00	35/20
	Std. De.	0/00	8/94	5/48	13/04	34/93	30/57
D15	Mean	0/00	72/00	22/00	44/00	66/00	40/80
	Std. De.	0/00	8/37	4/47	13/42	32/09	31/21
D18	Mean	0/00	80/00	26/00	52/00	74/00	46/40
	Std. De.	0/00	7/07	5/48	8/37	24/08	32/52
D21	Mean	0/00	82/00	30/00	60/00	80/00	50/40
	Std. De.	0/00	4/47	7/07	10/00	18/71	33/35
D24	Mean	0/00	84/00	40/00	64/00	80/00	53/60
	Std. De.	0/00	5/48	7/07	5/48	18/71	32/77
D27	Mean	0/00	88/00	46/00	68/00	82/00	56/80
	Std. De.	0.000	4.472	11.402	13.038	17.889	34.122
D30	Mean	0.000	94.000	50.000	72.000	82.000	59.600
	Std. De.	0.000	8.944	10.000	13.038	17.889	35.412

To assess the statistical significance of the observed differences among treatments over time, repeated-measures analysis of variance (ANOVA) was performed. Prior to analysis, the assumption of homogeneity of variances across groups was assessed using Levene’s test. The results of Levene’s test indicated that the variances among groups were not significantly different at the 0.05 level (p > 0.05), confirming that the homogeneity of variances assumption was met. Additionally, Mauchly’s test of sphericity was conducted to evaluate the assumption of sphericity for the within-subjects factor (time). Due to violation of the sphericity assumption, the Greenhouse-Geisser correction was applied. A summary of the results is presented in Table 4.

**Table 4:** Results of repeated-measures ANOVA for disease index, including within and between subjects effects.

	Source	SS	df	MS	F	Sig.	
Tests of Within-Subjects Effects	Time	34440.00	2.75	12547.60	84.09	0.00	0.81
	Time * Treatment	11880.00	10.98	1082.07	7.25	0.00	0.60
	Error(Time)	8191.11	54.89	149.21			
Tests of Between-Subjects Effects	Treatment	165344.00	4	41336.00	26.22	0.00	0.84
	Error	31528.89	20	1576.44			

*Note: SS: Sum of Squares; df: degrees of freedom; MS: Mean Square; Partial: Partial eta squared.

Mauchly’s test of sphericity indicated a violation of the sphericity assumption (Mauchly’s W = 0.002, approx. chi-square = 111.13, df = 35, p < 0.001). Therefore, the Greenhouse-Geisser correction (ε = 0.343) was applied to adjust the degrees of freedom. As shown in Table 2, repeated-measures ANOVA revealed a significant main effect of time on disease index (F = 84.09, Greenhouse-Geisser df = 2.75, p < 0.001, partial η² = 0.808), indicating that disease severity increased significantly over time regardless of treatment. The main effect of treatment was also significant (F = 26.22, df = 4, p < 0.001, partial η² = 0.840), demonstrating an overall significant difference among the five experimental groups. Furthermore, the time × treatment interaction was significant (F = 7.25, Greenhouse-Geisser df = 10.98, p < 0.001, partial η² = 0.592), indicating that the pattern of disease progression over time differed significantly among treatments. The large effect sizes (partial η² values ranging from 0.592 to 0.840) indicate high practical significance of these differences.

To further explore differences among treatments and to identify which specific treatments differed significantly from each other, pairwise comparisons were performed using the Bonferroni post-hoc test. Bonferroni-corrected pairwise comparisons revealed that the encapsulated oligoDNA treatment (Encaps oligos) significantly reduced disease index compared to the pathogen control (mean difference = −44.00, 95% CI: −70.40 to −17.61, p = 0.001). This treatment also showed a significant difference compared to the WPC treatment (mean difference = −42.67, 95% CI: −69.06 to −16.27, p = 0.001). However, the difference between encapsulated oligoDNA and non-encapsulated oligoDNA was not significant at the 0.05 level (mean difference = −21.11, p = 0.203). Importantly, the non-encapsulated oligoDNA treatment did not differ significantly from the pathogen control (mean difference = −22.89, p = 0.128), indicating that unencapsulated oligoDNAs alone had no significant inhibitory effect. Similarly, the WPC treatment (empty carrier) showed no significant difference from the pathogen control (mean difference = 1.33, p = 1.000), confirming that whey protein concentrate itself possesses no inherent antifungal activity. Collectively, these results demonstrate that encapsulation of oligoDNAs within the WPC matrix significantly enhances their antifungal efficacy against *V. dahliae*.

The effect of the different treatments on disease progression was also evaluated by calculating the Area Under the Disease Progress Curve (AUDPC) (S3 File, S3 Fig). Visual assessment revealed distinct differences among treatments. As anticipated, pathogen-inoculated (P) plants developed severe wilting and chlorosis, while non-inoculated controls (C) remained healthy. Notably, plants treated with non-encapsulated oligoDNAs displayed severe chlorosis and wilting, characteristic of *Verticillium* infection. In contrast, plants treated with the encapsulated oligoDNA formulation exhibited markedly reduced symptom development and normal growth of the plants. WPC treatment showed disease levels similar to the pathogen control, indicating the encapsulant alone provided no protective effect.

The AUDPC results were statistically analyzed using a one-way analysis of variance (ANOVA). The analysis revealed a highly significant difference among the treatment groups (F(4, 45) = 38.32, p < 0.001), indicating that the AUDPC values were not the same for all treatments. However, the assumption of homogeneity of variances was violated, as indicated by a significant Levene’s test (p < 0.001). Consequently, the robust Welch test was examined, but it could not be computed because the control group had zero variance. Therefore, post-hoc comparisons were conducted using the Games-Howell test, which is appropriate when variances are unequal.

The mean AUDPC for the untreated control group was 0.00 ± 0.00. In contrast, all treatments involving pathogen inoculation or application of oligoDNAs resulted in significantly higher AUDPC values compared to the control (p < 0.01 for all). Inoculation with the pathogen alone resulted in the highest mean disease level (88.95 ± 12.17), which was not significantly different from WPC treatment (84.75 ± 29.57, p = 0.993) but was significantly higher than all other treatment groups. Treatment with non-encapsulated oligoDNAs 159 + 166 yielded a mean AUDPC of 58.80 ± 20.43, which was significantly lower than the pathogen-only control (p = 0.009). Treatment with encapsulated oligoDNAs 159 + 166 resulted in a mean AUDPC of 32.85 ± 19.25, which was significantly lower than both the pathogen-only control (p < 0.001) and the non-encapsulated oligoDNAs (p = 0.030). Furthermore, the result of encapsulated oligoDNAs 159 + 166 treatment was not significantly different from the non-encapsulated treatment (p = 0.061). These results demonstrate that while both oligoDNA formulations significantly reduced disease severity compared to pathogen inoculation alone, the encapsulated formulation was the most effective, reducing the AUDPC by approximately 63% relative to the pathogen-only control.

## Discussion

This study evaluated the antifungal efficacy of whey protein concentrate (WPC) microencapsulated double-stranded DNA oligonucleotides mimicking microRNA166 (miR166) and microRNA159 (miR159) for controlling the soil-borne fungal pathogen *V. dahliae*. The oligonucleotides were encapsulated using electrospraying technology with WPC as a biodegradable carrier, aiming to enhance their stability and enable controlled release. The differences in particle size measurements arise because the techniques analyze the particles in fundamentally different states. SEM and TM measure the physical diameter of *dry, solid* particles. In contrast, DLS measures the *hydrodynamic diameter* of particles in an aqueous suspension. Because our WPC microcapsules are hydrophilic and can swell in water, their size in suspension (measured by DLS) is inherently larger than their size in a dehydrated state (measured by SEM/TEM). Furthermore, DLS is an intensity-based technique that is highly sensitive to the presence of even a few large aggregates, which skews the average to higher values, while microscopy allows us to visualize and measure individual primary particles [55,56].

The PDI values reported reflect the inherent characteristics of electrospraying complex biopolymers like WPC, which naturally results in a broader size distribution. Achieving perfect monodispersity is challenging with biological macromolecules. Importantly, for controlled-release applications in agriculture, a moderately heterogeneous particle population can be advantageous. A mixture of particle sizes can lead to a more sustained and multi-phasic release profile, potentially offering prolonged protection to the plant compared to a monodisperse system [57,58].

The results demonstrate that oligoDNA159 and oligoDNA166 significantly inhibit *V. dahliae* growth in vitro and reduce disease severity in planta. This antifungal effect is likely mediated by an RNA interference (RNAi) mechanism, consistent with gene-silencing activity of essential fungal virulence factors. These findings align with previous work showing that cotton plants express miR166 and miR159 upon *V. dahliae* infection and transfer these microRNAs into fungal hyphae to silence pathogenicity genes *Clp-1* and *HiC-15*, respectively [32]. The reduction in fungal colony growth observed here mirrors the phenotype reported for mutant strains with inactivated *Clp-1* or *HiC-15* genes, corroborating the targeted consistent with gene-silencing activity hypothesis. However, the absence of scrambled or mismatch oligonucleotide controls limits definitive conclusions regarding sequence specificity.

Encapsulation of oligonucleotides in WPC microcapsules enhanced their antifungal efficacy compared to free oligos, likely by protecting them from environmental degradation and facilitating controlled, sustained release. This improvement supports the suitability of WPC as an encapsulant material due to its excellent electrospraying properties and biocompatibility [28–31]. The lack of antifungal activity in microcapsules without oligonucleotides confirms that the inhibitory effect is attributable to the functional DNA sequences rather than the protein matrix alone.

Fluorescence microscopy revealed that Verticillium hyphae interact with and potentially internalize Cy3-labeled oligoDNAs, as evidenced by localized fluorescence clusters and punctate intracellular-like signals, likely via an actin- and clathrin-dependent endocytic pathway consistent with recent live-cell imaging studies on *V. dahliae* RNA internalization [45,59]. While epifluorescence alone cannot definitively distinguish surface binding from true association consistent with uptake, the functional evidence for intracellular delivery is compellingly substantiated by three complementary observations: significant and sequence-specific downregulation of target genes (*HiC-15* and *Clp-1*) quantified by RT-qPCR, which requires oligonucleotide access to the cytoplasmic RNA-induced silencing complex; reduced fungal colony growth rates following oligoDNA treatment; and decreased pathogenicity in plant infection assays. Collectively, these molecular and phenotypic data, alongside similar patterns of RNA uptake reported in other filamentous fungi [13,14,60], provide robust evidence that oligonucleotides were internalized and biologically active, supporting the concept of RNA-based fungicidal strategies relying on pathogen uptake for gene silencing. Future studies employing confocal microscopy with z-stack analysis and quenching assays will provide direct visual confirmation of intracellular localization and further elucidate this promising mechanism [61,62].

The release of oligoDNA from WPC-based microcapsules is hypothesized to occur through a combination of diffusion and protein matrix degradation. WPC microcapsules are hydrophilic and swell upon exposure to aqueous environments (such as soil moisture or plant surfaces), increasing the pore size within the protein matrix and allowing entrapped oligoDNA molecules to diffuse outward gradually [58]. Additionally, WPC is a biodegradable protein susceptible to proteolytic enzymes produced by soil microorganisms and plant root exudates. Enzymatic degradation of the WPC matrix would further facilitate the controlled and sustained release of the encapsulated oligoDNA over time [28,31]. The BET analysis in our study confirmed the micro-mesoporous structure of the microcapsules (pore sizes 1–3 nm), which supports the feasibility of diffusion-based release. Furthermore, the TGA and DSC data demonstrated that oligoDNA incorporation enhanced the thermal stability of the microcapsules, suggesting molecular interactions between WPC and oligoDNA that may modulate release kinetics. Future studies could employ in vitro release assays under simulated environmental conditions to quantitatively characterize the release profile.

This study’s results corroborate the promise of DNA oligonucleotides as a cost-effective and stable alternative to dsRNA for spray-induced gene silencing approaches in plant disease management. The demonstrated suppression of Verticillium wilt symptoms underlines the potential utility of oligo encapsulation via WPC microcapsules as an environmentally friendly strategy to reduce reliance on conventional fungicides. Future work should optimize oligonucleotide formulations for improved uptake, stability, and field application, as well as explore the silencing efficiency against additional pathogenic targets.

In conclusion, this research provides strong evidence that WPC microencapsulated oligoDNA159 and oligoDNA166 effectively inhibit *V. dahliae*, and the observed transcript suppression is consistent with gene-silencing activity. Oligonucleotide encapsulation improves stability and bioavailability, offering a promising platform for the development of sustainable and targeted antifungal biocontrol agents.

## Supporting information

S1 Fig Fluorescence microscopy image of *V. dahliae* treated with Cy3-labeled oligo-DNA. A) Fluorescence microscopy image (40×) showing bright fluorescent puncta indicate successful attachment and/or intracellular uptake of Cy3-conjugated molecules, demonstrating the fungus’s capacity to internalize exogenous oligo-DNA cargo. B) Low-magnification fluorescence image (10×) showing dense clusters of bright fluorescent puncta within fungal biomass, indicating localized accumulation of Cy3-labeled oligonucleotides at the tissue/mass level.
(TIF)

S2 Fig Effect of different treatments on Verticillium colony growth. Quantitative analysis of mean colony area (cm²) for each treatment group. Bars represent mean ± SD (n = 5 per group). Treatments with different lowercase letters are significantly different according to the one-way ANOVA analysis; Games-Howell post-hoc test (p < 0.05).
(TIF)

S3 Fig Effect of different treatments on disease suppression in tomato seedlings and Area Under the Disease Progress Curve (AUDPC). A) Visual assessment of Verticillium wilt symptoms on tomato plants one month after treatment. Representative plants are shown for the non-encapsulated oligoDNAs 159 + 166, encapsulated oligoDNAs 159 + 166, WPC encapsulant without oligoDNA, C—Non-inoculated negative control, and P—Pathogen-only positive control. B) Mean disease index (± SD) across different treatments in different time points. C) Showes AUDPC measures for each treatment. Bars represent mean ± standard deviation (n = 10). Error bars indicate standard deviation of the mean (SD). Treatments with different lowercase letters are significantly different according to the one-way ANOVA analysis; Games-Howell post-hoc test (p < 0.05).
(TIF)

S1 File Colony growth area raw data. (DOCX)

S2 File The Disease Index (DI) dataset and statistic analysis. (XLSX)

S3 File Area Under the Disease Progress Curve (AUDPC) dataset. (DOCX)
