# Supplementary figures and images for: Antifungal efficacy of microencapsulated oligoDNAs through whey protein concentrate (WPC) as coated protein against Verticillium dahliae
Source: PLoS One. 2026 May 27;21(5):e0349566. doi: 10.1371/journal.pone.0349566 (PMC13215511; doi:10.1371/journal.pone.0349566)

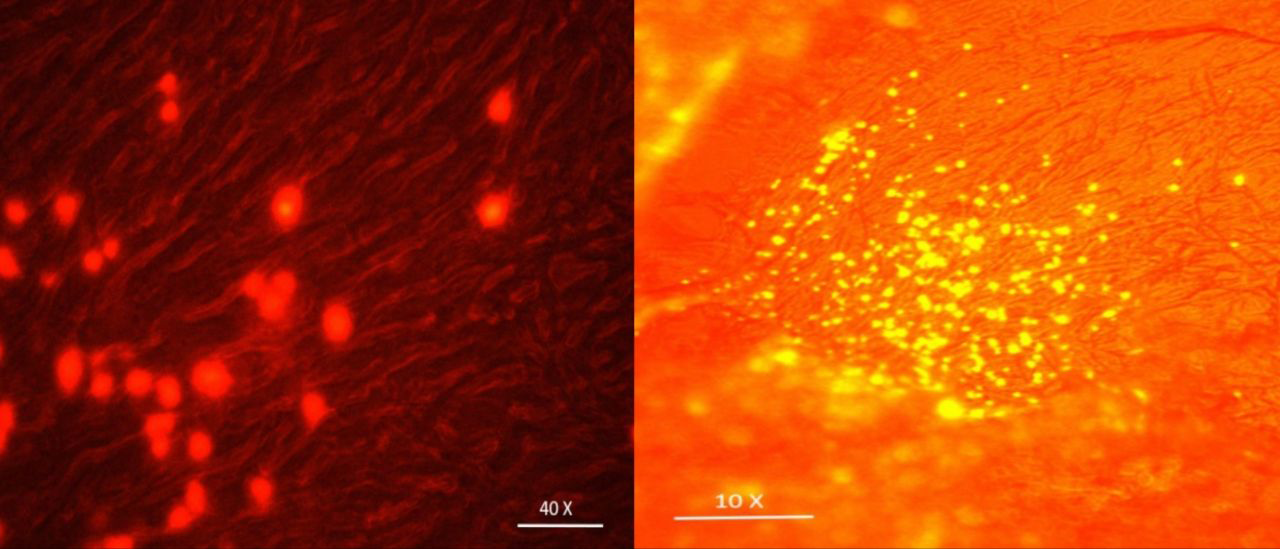

Supplement: S1 Fig — A) Fluorescence microscopy image (40×) showing bright fluorescent puncta indicate successful attachment and/or intracellular uptake of Cy3-conjugated molecules, demonstrating the fungus’s capacity to internalize exogenous oligo-DNA cargo. B) Low-magnification fluorescence image (10×) showing dense clusters of bright fluorescent puncta within fungal biomass, indicating localized accumulation of Cy3-labeled oligonucleotides at the tissue/mass level. (TIF) [file pone.0349566.s001.tif]

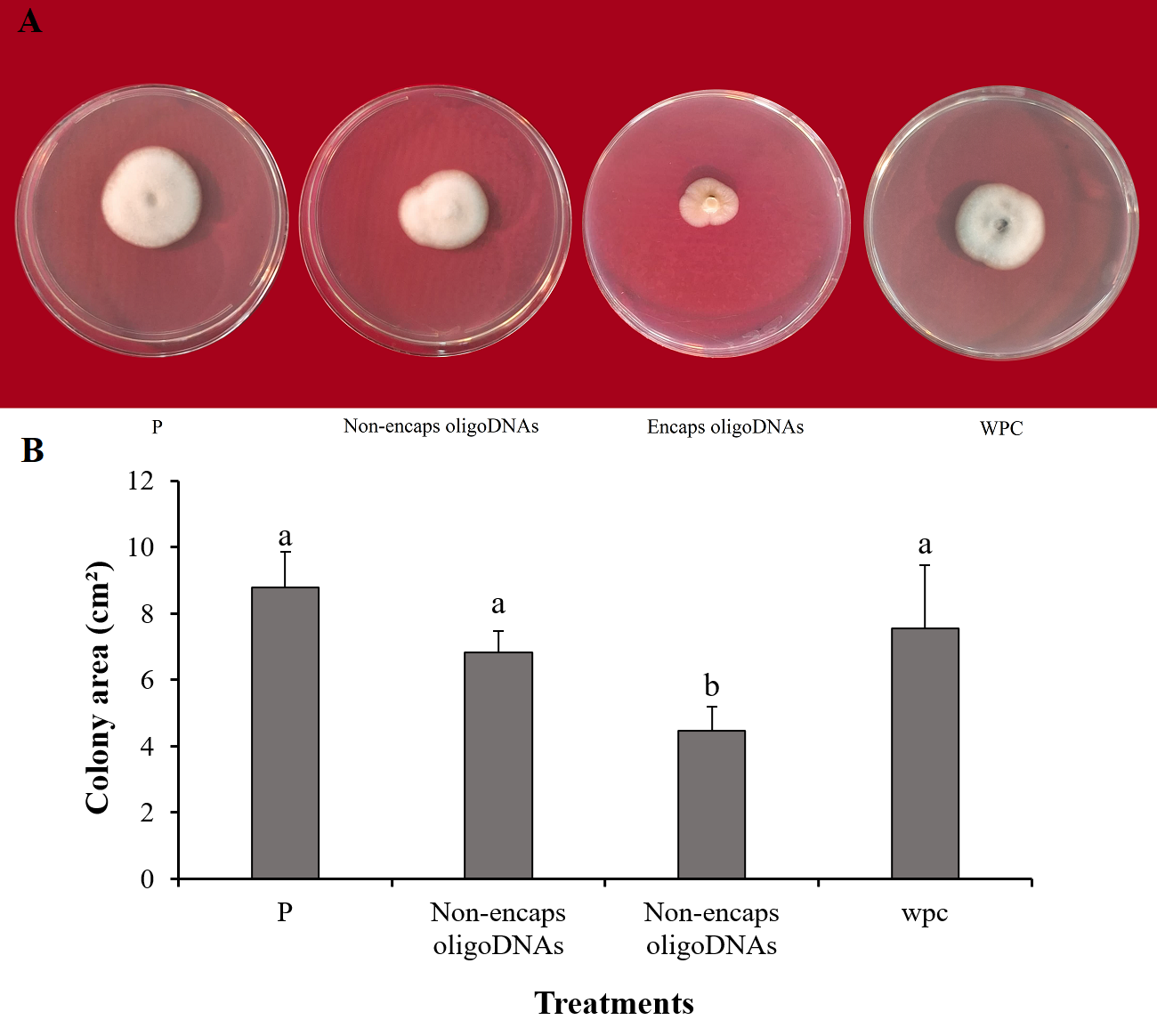

Supplement: S2 Fig — Quantitative analysis of mean colony area (cm²) for each treatment group. Bars represent mean ± SD (n = 5 per group). Treatments with different lowercase letters are significantly different according to the one-way ANOVA analysis; Games-Howell post-hoc test (p < 0.05). (TIF) [file pone.0349566.s002.tif]

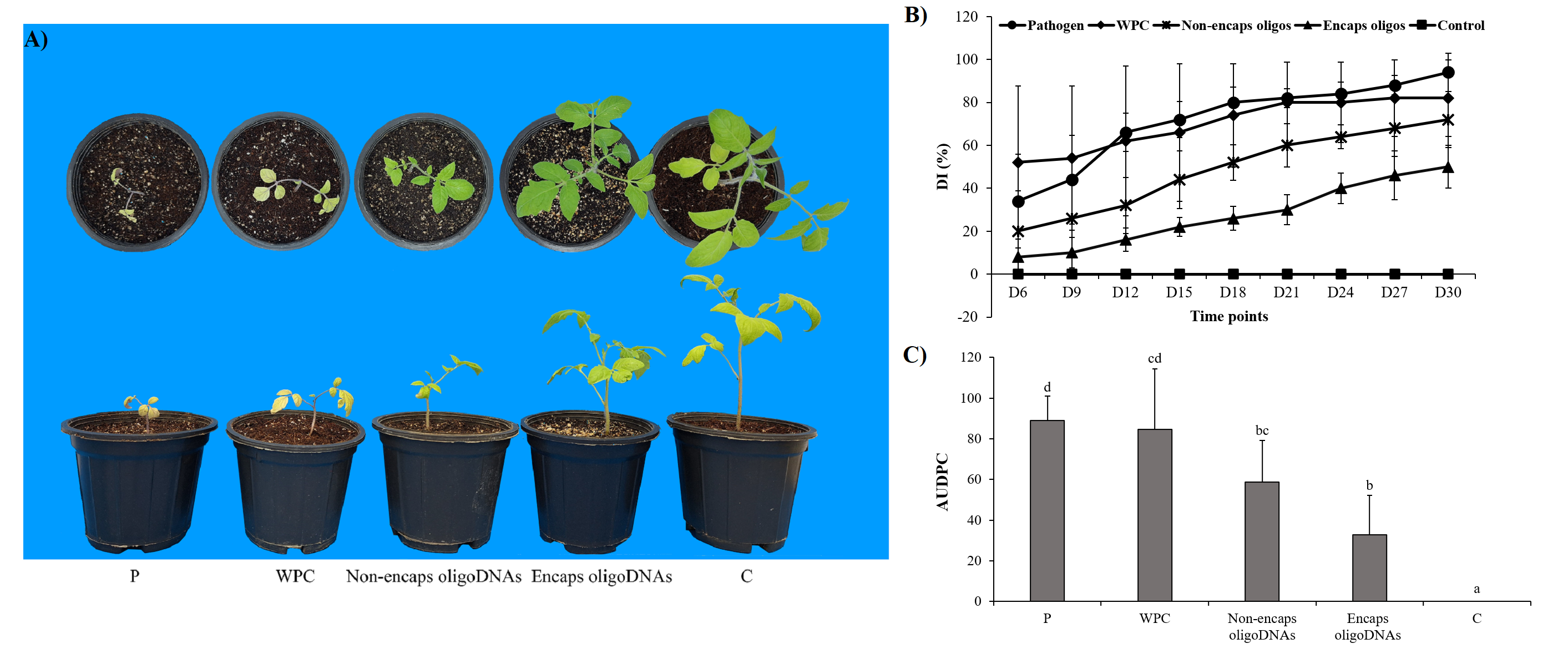

Supplement: S3 Fig — A) Visual assessment of Verticillium wilt symptoms on tomato plants one month after treatment. Representative plants are shown for the non-encapsulated oligoDNAs 159 + 166, encapsulated oligoDNAs 159 + 166, WPC encapsulant without oligoDNA, C—Non-inoculated negative control, and P—Pathogen-only positive control. B) Mean disease index (± SD) across different treatments in different time points. C) Showes AUDPC measures for each treatment. Bars represent mean ± standard deviation (n = 10). Error bars indicate standard deviation of the mean (SD). Treatments with different lowercase letters are significantly different according to the one-way ANOVA analysis; Games-Howell post-hoc test (p < 0.05). (TIF) [file pone.0349566.s003.tif]
