# Supplementary material for: Antifungal efficacy of microencapsulated oligoDNAs through whey protein concentrate (WPC) as coated protein against Verticillium dahliae
Source: PLoS One. 2026 May 27;21(5):e0349566. doi: 10.1371/journal.pone.0349566 (PMC13215511; doi:10.1371/journal.pone.0349566)
Supplement: S1 File — (DOCX) [file pone.0349566.s004.docx]

| Colony Area (cm²) | Treatment |
| --- | --- |
| 9/07 | pathogen |
| 9/62 | pathogen |
| 8/55 | pathogen |
| 9/62 | pathogen |
| 7/07 | pathogen |
| 7/07 | Non-encaps oligoDNA |
| 6/91 | Non-encaps oligoDNA |
| 6/31 | Non-encaps oligoDNA |
| 6/15 | Non-encaps oligoDNA |
| 7/72 | Non-encaps oligoDNA |
| 5/31 | Encaps oligoDNA |
| 3/46 | Encaps oligoDNA |
| 4/15 | Encaps oligoDNA |
| 4/52 | Encaps oligoDNA |
| 4/91 | Encaps oligoDNA |
| 5/72 | wpc |
| 9/62 | wpc |
| 6/15 | wpc |
| 9/62 | wpc |
| 6/60 | wpc |
