# Supplementary material for: Antifungal efficacy of microencapsulated oligoDNAs through whey protein concentrate (WPC) as coated protein against Verticillium dahliae
Source: PLoS One. 2026 May 27;21(5):e0349566. doi: 10.1371/journal.pone.0349566 (PMC13215511; doi:10.1371/journal.pone.0349566)
Supplement: S3 File — (DOCX) [file pone.0349566.s006.docx]

0: No symptoms, 1: Mild leaf wilting, 2: Yellowing of some leaves, 3: Moderate wilting and significant yellowing, 4: Severe wilting and stunting, 5: Plant death.

| Control | No. | D6 | D9 | D12 | D15 | D18 | D21 | D24 | D27 | D30 | AUDPC |
| --- | --- | --- | --- | --- | --- | --- | --- | --- | --- | --- | --- |
|  | 1 | 0 | 0 | 0 | 0 | 0 | 0 | 0 | 0 | 0 | 0 |
|  | 2 | 0 | 0 | 0 | 0 | 0 | 0 | 0 | 0 | 0 | 0 |
|  | 3 | 0 | 0 | 0 | 0 | 0 | 0 | 0 | 0 | 0 | 0 |
|  | 4 | 0 | 0 | 0 | 0 | 0 | 0 | 0 | 0 | 0 | 0 |
|  | 5 | 0 | 0 | 0 | 0 | 0 | 0 | 0 | 0 | 0 | 0 |
|  | 6 | 0 | 0 | 0 | 0 | 0 | 0 | 0 | 0 | 0 | 0 |
|  | 7 | 0 | 0 | 0 | 0 | 0 | 0 | 0 | 0 | 0 | 0 |
|  | 8 | 0 | 0 | 0 | 0 | 0 | 0 | 0 | 0 | 0 | 0 |
|  | 9 | 0 | 0 | 0 | 0 | 0 | 0 | 0 | 0 | 0 | 0 |
|  | 10 | 0 | 0 | 0 | 0 | 0 | 0 | 0 | 0 | 0 | 0 |
|  |  |  |  |  |  |  |  |  |  |  |  |
|  |  |  |  |  |  |  |  |  |  |  |  |
| Pathogen | No. | D6 | D9 | D12 | D15 | D18 | D21 | D24 | D27 | D30 | AUDPC |
|  | 1 | 2 | 2 | 3 | 4 | 4 | 4 | 4 | 4 | 5 | 85/5 |
|  | 2 | 1 | 1 | 3 | 4 | 4 | 4 | 4 | 5 | 5 | 84 |
|  | 3 | 2 | 2 | 3 | 3 | 4 | 4 | 4 | 4 | 4 | 88/5 |
|  | 4 | 1 | 2 | 3 | 3 | 4 | 4 | 4 | 5 | 5 | 84 |
|  | 5 | 2 | 2 | 3 | 4 | 4 | 4 | 4 | 4 | 4 | 84 |
|  | 6 | 1 | 1 | 3 | 3 | 3 | 4 | 4 | 4 | 4 | 73/5 |
|  | 7 | 1 | 3 | 4 | 4 | 4 | 4 | 4 | 4 | 5 | 90 |
|  | 8 | 0 | 1 | 3 | 3 | 4 | 4 | 5 | 5 | 5 | 94/5 |
|  | 9 | 2 | 3 | 3 | 3 | 4 | 4 | 4 | 4 | 5 | 85/5 |
|  | 10 | 5 | 5 | 5 | 5 | 5 | 5 | 5 | 5 | 5 | 120 |
|  |  |  |  |  |  |  |  |  |  |  |  |
|  |  |  |  |  |  |  |  |  |  |  |  |
| Encaps oligos | No. | D6 | D9 | D12 | D15 | D18 | D21 | D24 | D27 | D30 | AUDPC |
|  | 1 | 0 | 0 | 0 | 0 | 0 | 0 | 1 | 1 | 1 | 7/5 |
|  | 2 | 2 | 2 | 2 | 2 | 3 | 3 | 4 | 4 | 4 | 69 |
|  | 3 | 0 | 0 | 0 | 0 | 1 | 1 | 1 | 2 | 2 | 18 |
|  | 4 | 0 | 1 | 2 | 2 | 2 | 2 | 3 | 4 | 4 | 54 |
|  | 5 | 0 | 0 | 0 | 0 | 0 | 1 | 1 | 1 | 2 | 12 |
|  | 6 | 0 | 0 | 2 | 2 | 2 | 2 | 2 | 2 | 2 | 39 |
|  | 7 | 0 | 0 | 0 | 1 | 1 | 1 | 2 | 2 | 2 | 24 |
|  | 8 | 1 | 1 | 1 | 1 | 1 | 1 | 2 | 2 | 2 | 31/5 |
|  | 9 | 1 | 1 | 1 | 2 | 2 | 2 | 2 | 3 | 3 | 45 |
|  | 10 | 0 | 0 | 0 | 1 | 1 | 2 | 2 | 2 | 3 | 28/5 |
|  |  |  |  |  |  |  |  |  |  |  |  |
|  |  |  |  |  |  |  |  |  |  |  |  |
| Non-encaps oligos | No. | D6 | D9 | D12 | D15 | D18 | D21 | D24 | D27 | D30 | AUDPC |
|  | 1 | 1 | 1 | 1 | 1 | 2 | 3 | 3 | 3 | 4 | 49/5 |
|  | 2 | 3 | 3 | 3 | 4 | 4 | 4 | 4 | 4 | 4 | 88/5 |
|  | 3 | 0 | 1 | 1 | 2 | 2 | 3 | 3 | 4 | 4 | 54 |
|  | 4 | 4 | 4 | 4 | 4 | 4 | 4 | 4 | 5 | 5 | 100/5 |
|  | 5 | 0 | 0 | 0 | 1 | 2 | 2 | 3 | 3 | 3 | 37/5 |
|  | 6 | 0 | 1 | 2 | 2 | 2 | 3 | 3 | 3 | 3 | 52/5 |
|  | 7 | 0 | 0 | 2 | 3 | 3 | 3 | 3 | 3 | 3 | 55/5 |
|  | 8 | 1 | 1 | 1 | 2 | 2 | 3 | 3 | 3 | 3 | 51 |
|  | 9 | 0 | 0 | 0 | 1 | 2 | 2 | 3 | 3 | 3 | 37/5 |
|  | 10 | 1 | 2 | 2 | 2 | 3 | 3 | 3 | 3 | 4 | 61/5 |
|  |  |  |  |  |  |  |  |  |  |  |  |
|  |  |  |  |  |  |  |  |  |  |  |  |
| WPC | No. | D6 | D9 | D12 | D15 | D18 | D21 | D24 | D27 | D30 | AUDPC |
|  | 1 | 1 | 1 | 1 | 1 | 2 | 3 | 3 | 3 | 3 | 48 |
|  | 2 | 2 | 2 | 2 | 2 | 3 | 4 | 4 | 4 | 4 | 72 |
|  | 3 | 1 | 1 | 2 | 2 | 3 | 3 | 3 | 3 | 3 | 57 |
|  | 4 | 1 | 2 | 2 | 3 | 3 | 3 | 3 | 3 | 3 | 63 |
|  | 5 | 1 | 1 | 1 | 2 | 3 | 3 | 3 | 4 | 4 | 58/5 |
|  | 6 | 2 | 2 | 3 | 3 | 3 | 4 | 4 | 4 | 4 | 78 |
|  | 7 | 4 | 4 | 5 | 5 | 5 | 5 | 5 | 5 | 5 | 115/5 |
|  | 8 | 4 | 4 | 5 | 5 | 5 | 5 | 5 | 5 | 5 | 115/5 |
|  | 9 | 5 | 5 | 5 | 5 | 5 | 5 | 5 | 5 | 5 | 120 |
|  | 10 | 5 | 5 | 5 | 5 | 5 | 5 | 5 | 5 | 5 | 120 |
